# Malignant peripheral nerve sheath tumour in a sow

**DOI:** 10.1186/s13028-015-0150-y

**Published:** 2015-09-25

**Authors:** Talita P. Resende, Carlos E. R. Pereira, Fabio A. Vannucci, Fernando S. Araujo, José Lúcio dos Santos, Geovanni D. Cassali, Karine A. Damasceno, Roberto M. C. Guedes

**Affiliations:** Department of Veterinary Clinic and Surgery, Veterinary School, Universidade Federal de Minas Gerais, Av. Antônio Carlos, 6627, Pampulha, Belo Horizonte, MG 31270-901 Brazil; Laboratório de Microbiologia Veterinária, Microvet, Rua Joaquim Lopes de Faria, 730, Viçosa, MG 36570-000 Brazil; Praça Coronel Amantino 50, Piranga, MG 36480-000 Brazil; Department of General Pathology, Institute of Biological Sciences, Universidade Federal de Minas Gerais, Av. Antônio Carlos, 6627, Pampulha, Belo Horizonte, MG 31270-901 Brazil

**Keywords:** Swine, Peripheral nerve sheath tumor, Schwannoma, Lung

## Abstract

Nodular lung lesions in swine are frequently due to abscesses or granulomatous pneumonia. Although tumours are rarely reported in modern pig farming, they should be considered as a differential diagnosis when nodular lung lesions are found. A first-parity sow exhibiting respiratory signs was euthanized. Several whitish firm nodules, not encapsulated, ranging in diameter from 0.5 to 5 cm were present in all lung lobes. Microscopically, the nodules were composed of dense neoplastic cells, mainly in Antoni types A and B patterns, infiltrative and with development of emboli. All neoplastic cells stained positively by immunohistochemistry for vimentin and S-100 protein, with variable immunostaining for glial fibrillary acidic protein and stained negative for cytokeratin. Based on the gross, histological and immunohistochemical features, the tumor was diagnosed as malignant peripheral nerve sheath tumour.

## Background

Gross nodular lung lesions in pigs are frequently associated with abscesses [1] or granulomatous pneumonia [2] but may also be caused by other conditions such as neoplasms. Although neoplasms probably occur more frequently in swine than reflected in the scientific literature, based on person reports of lymphomas and melanoms in different age of pigs, neoplasm are still rather uncommon compared to other lesions. This may be due to the low average age of the population and porcine neoplasms are generally less prevalent than in animal species with a longer life span [3].

Malignant peripheral nerve sheath tumours (MPNSTs) (former “schwannomas”) are relatively common in humans [4] and cattle [5] but rarely reported in other species [6]. According to available reports, there is only one description of a PNST in swine, which was an incidental finding in the skin of a pig at slaughter [7].

This report describes the gross and histopathological lesions as well as histochemical and immunohistochemical findings in a case of a porcine pulmonary MPNST.

## Case presentation

**Fig. 1:**
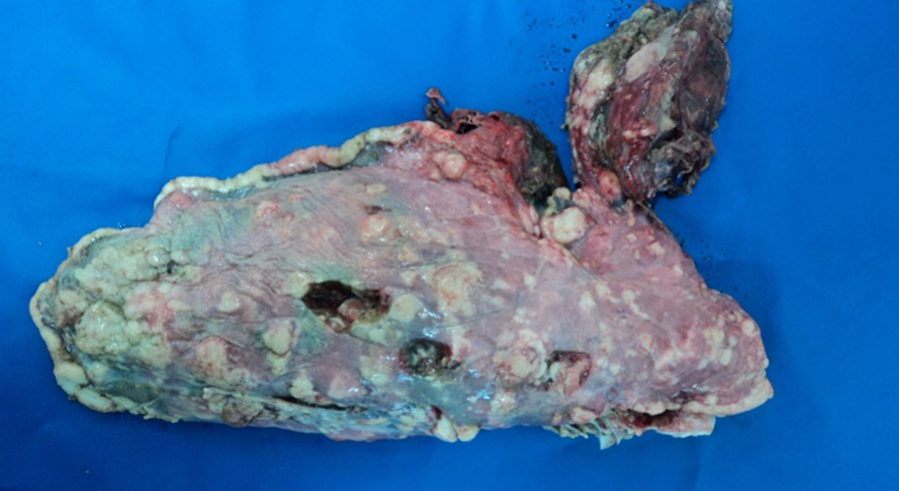
Multifocal whitish firm not encapsulated nodules with a diameter ranging from 0.5 to 5 cm in the lung of a sow

A first-parity sow with a history of progressive weight loss, anorexia, lethargy and a cough was humanely euthanized due to severe respiratory distress. The sow was necropsied in the field by the local veterinarian and several whitish firm not encapsulated nodules, ranging in diameter from 0.5 to 5 cm, were present int all lung lobes (Fig. 1) and mediastinal lymph nodes were enlarged. No other lesions were observed.

The lung and lymph nodes were submitted to the Department of General Pathology, Universidade Federal de Minas Gerais. Specimens were fixed in 10 % neutral buffered formalin, processed routinely and embedded in paraffin. Sections of 4 µm were prepared and stained with haematoxylin-eosin (HE). Additionally, special stainings were performed using Masson’s and Gomori’s trichrome to identify connective tissues and muscle cells.

Immunohistochemistry was performed for cytokeratin (CK AE1/AE3, Dako 1:100), vimentin (V9, Dako, Glostrup, Denmark, 1:50), S-100 (polyclonal, Dako, 1:200) and glial fibrillary acid protein (GFAP) (Sigma-Aldrich, 1:500). Antigen retrieval solution was applied (DakoCytomation) at pH 6.0 in a water bath at 98 °C for 20 min. GFAP was performed using a citrate buffer at pH 7.2 for 30 min at 98 °C. Endogenous peroxidase was blocked with 3 % of hydrogen peroxide in methanol for 30 min, followed by treatment with Protein Block Serum-Free (DakoCytomation). Slides were incubated with primary antibodies CK AE1/AE3, vimentin and S-100 for 1 h at room temperature and GFAP overnight (16 h) at 4 °C. A polymer detection system was used in the immunohistochemical procedure with a secondary antibody (ADVANCE HRP-ready to use, DakoCytomation) and the chromogen 3,3′diaminobenzidine tetrahydrocholride-hydrogen peroxide. Samples were counterstained with Mayer’s haematoxylin.

**Fig. 2:**
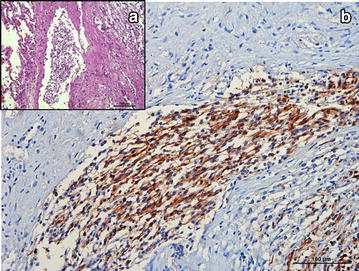
Photomicrograph of a porcine pulmonary malignant peripheral nerve sheet tumor. **a** Neoplastic cells in linfatic vassel, HE. **b** GFAP immunolabeling of neoplastic emboli

**Fig. 3:**
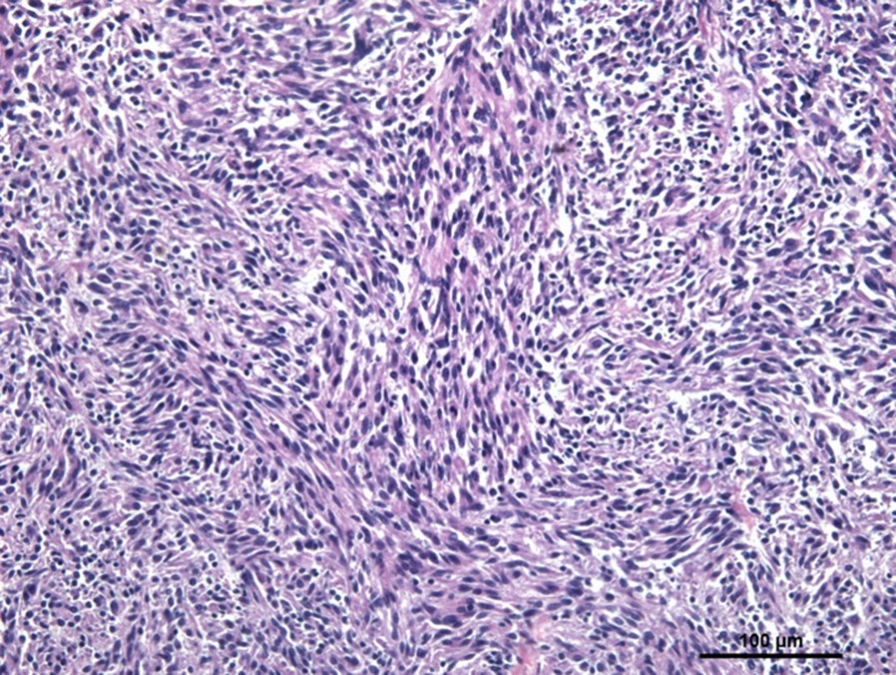
Photomicrograph showing neoplastic spindled-shaped cells arranged in short interlacing fascicles (Antoni type A pattern) interspersed by small round cells in a loosely textured matrix (Antoni type B). Porcine pulmonary malignant peripheral nerve sheet tumor, HE

Microscopically, the nodules were composed of dense neoplastic cells that infiltrated the surrounding lung tissue and invaded the wall and lumen of lymphatic vessels (Fig. 2). Spindle-shaped neoplastic cells, supported by a dense connective tissue layer¸ were arranged in small dense palisades, in bundles of different directions or in whorls (Antoni type A pattern) (Fig. 3). In Antoni type B type areas, round or oval neoplastic cells proliferated haphazardly within a loosely textured myxoid matrix, occasionally having cytoplasmic vacuoles. No Verocay bodies were found. Tumor cells showed marked anisocytosis, moderate anisokaryosis and central rounded nuclei with loose chromatin. There was a moderate mitotic index. Multifocal necrotic areas were also found, occasionally accompanied by a neutrophilic infiltrate. No lesions were found in mediastinal lymph nodes.

**Fig. 4:**
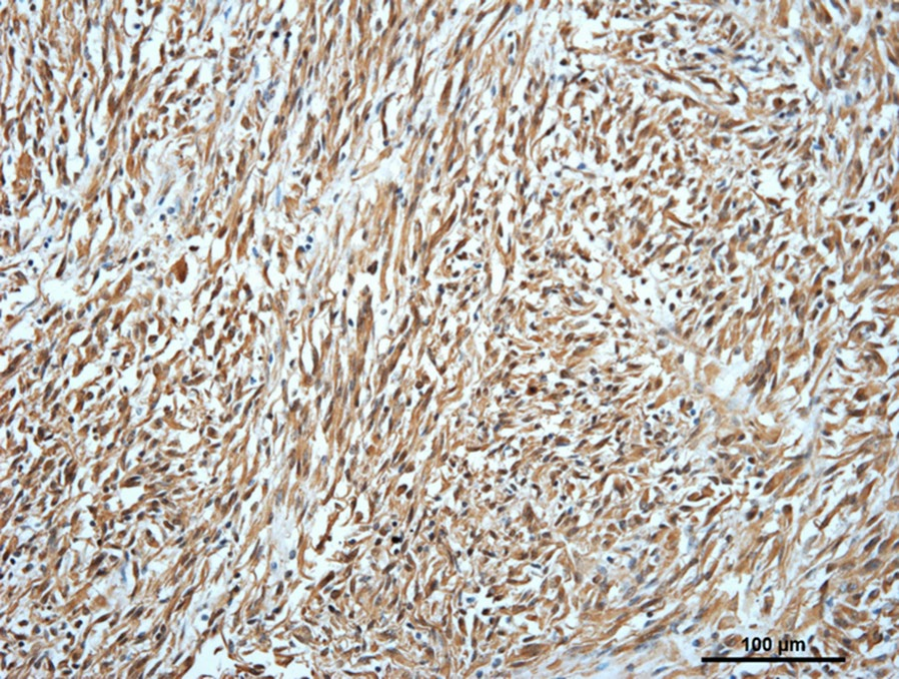
Photomicrograph showing positive vimentin immunolabeling of spindle-shaped and round neoplastic cells. Porcine pulmonary malignant peripheral nerve sheet tumor

Examination of Masson’s and Gomori trichrome stained sections did not demonstrate positive staining neither for connective tissue nor for muscle cells in neoplastic areas. Almost all of the neoplastic cells stained positively for vimentin (Fig. 4) and S-100 protein and negative for cytokeratin. Staining for GFAP showed variable immunostaining in the neoplastic cells, i.e. weak and sparse in Antoni type B patterns and moderate to strong in spindle-shaped cells from the Antoni type A patterns, including intravascular located neoplastic cells (Fig. 2). Based on histomorphological characteristics and the immunohistochemical staining results, a diagnosis of a MPNST was made.

MPNSTs originate from nervous cells, and their classification is frequently contentious. Schwannomas are one type of peripheralnerve sheath tumor (PNST) and was the classic nomenclature used for tumors that were specifically derived from myelinating cells [8, 9]. The histologic pattern for this tumor in animals was based on the human pattern [10]. As there is no specific immunohistochemical staining for MPNSTs, a final diagnosis is often difficult [11]. However, strong positivity for vimentin, S-100 and a variable GFAP labelling (mainly in Antoni type A pattern) are frequently reported [11–16].

Due to the anatomy of the peripheral nervous system, PNSTs can be found in various sites. In human medicine, PNSTs are the most frequent benign neurogenic tumor and are rarely found in a malignant form. The most common sites of PNSTs in humans are the head, neck and limbs [15]. Although the tumor is often an incidental finding in cattle [14], Peek et al. [17] reported chronic tympanism and paresis in a cow associated with a MPNST and Nielsen et al. [18] related clinical signs in three cows to the presence of PNSTs in the vertebral canal. Similarly, severe complications associated with MPNSTs have been found in horses. Quinn et al. [19] described this tumor in the heart, which caused circulatory collapse in a cob cross equine, and Kirchhof et al. [20] described an acute episode of colic caused by numerous MPNSTs in the intestinal wall. MPNSTs in dogs have been reported in different sites, such as the testis [21], trigeminal nerve [22], nerve plexus, thorax and abdomen [4]. Cats are less frequently affected; however, MPNSTs have been reported as cutaneous and subcutaneous nodules [4] or in the limbs [15].

In pigs, Tanimoto and Ohtsuki [7] described a solitary mass in the dermis, with Antoni types A and B patterns. Most of cells showed positive labeling to S-100 and vimentin, in addition to cytokeratin labeling in peripherally located cells. Based on ultrastructural studies, these authors concluded that it was a cutaneous plexiform PNST.

## Conclusion

In the present case, histologic features characterized by Antoni types A and B patterns with neoplastic cell invading lymphatic vessels and being associated with marked positive staining for S-100 and vimentin and variable staining for GFAP, supported the diagnosis of a MPNST. This is the first report of of porcine pulmonary MPNST.

## Abbreviations

CK: cytokeratin
GFAP: glial fibrilar acid protein
HE: haematoxilin-eosin
MPNST: malignant peripheral nerve sheath tumor
PNST: peripheral nerve sheath tumor
V9: vimentin

## References

[1] Alberton GC, Mores MAS (2009). Interpretation of injuries in the slaughter as a tool for diagnosis of respiratory diseases in pigs. Acta Sci Vet.

[2] Thoen CO. Tuberclosis. In: Straw BE, Zimmerman JJ, D’Allaire S, Taylor DJ, editors. Diseases of Swine, 9th ed. Ames: Blackwell Publishing Professional; 2006. p. 807–16.

[3] Cameron RDA. Diseases of the skin. In: Straw BE, Zimmerman JJ, D’Allaire S, Taylor DJ, editors. Diseases of Swine, 9th ed. Ames: Blackwell Publishing Professional; 2006. p. 196.

[4] Franks AJ (1985). Epithelioid neurilemmoma of the trigeminal nerve: an immunohistochemical and ultrastructural study. Histopathology.

[5] Grossi AB, Agerholm JS, Christensen K, Jensen HE, Leifsson PS, Bendixen C (2014). A hereditary disposition for bovine peripheral nerve sheath tumors in Danish Holstein cattle. Acta Vet Scand.

[6] Stoica G, Tasca SI, Kim HT (2001). Point mutation of neu oncogene in animal peripheral nerve sheath tumors. Vet Pathol.

[7] Tanimoto T, Ohtsuki Y (1993). Cutaneous plexiform schwannoma in a pig. J Comp Pathol.

[8] Goldschmidtm MH, Hendrick MJ. Benign peripheral nerve sheath tumor (neurofibroma, schwannoma). In: Meuten DJ, editor. Tumors in Domestic Animals, 4th ed. Ames: Iowa State University Press; 2002. p. 95–6.

[9] Joshi R (2012). Learning from eponyms: Jose Verocay and Verocay bodies, Antoni A and B areas, Nils Antoni and Schwannomas. Indian Dermatol Online J.

[10] Enzinger FM, Weiss SW. Benign tumors of peripheral nerves. In: Soft Tissue Tumors, 3rd ed. St Louis: Mosby-Year Book; 1995. p. 821–88.

[11] Kawahara E, Oda Y, Ooi A, Katsuda S, Nakanishi I, Umeda S (1988). Expression of glial fibrillary acidic protein (GFAP) in peripheral nerve sheath tumors. A comparative study of immunoreactivity of GFAP, vimentin, S-100 protein, and neurofilament in 38 schwannomas and 18 neurofibromas. Am J Surg Pathol.

[12] Sawamoto O, Yamate J, Kuwamura M, Hagiwara R, Kurisu KA (1999). Canine peripheral nerve sheath tumor including peripheral nerve fibers. J Vet Med Sci..

[13] Chijiwa K, Uchida K, Tateyama S (2004). Immunohistochemical evaluation of canine peripheral nerve sheath tumors and other soft tissue sarcomas. Vet Pathol.

[14] Beytut E (2006). Multicentric malignant schwannoma in a crossbred cow. J Comp Pathol.

[15] Schulman FY, Johnson TO, Facemire PR, Fanburg-Smith JC (2009). Feline peripheral nerve sheath tumors: histologic, immunohistochemical, and clinicopathologic correlation (59 tumors in 53 cats). Vet Pathol.

[16] Bogaert L, Van Heerden M, De Cock HEV, Martens A, Chiers K (2011). Molecular and immunohistochemical distinction of equine sarcoid from schwannoma. Vet Pathol.

[17] Peek SF, Del Piero F, Rebhun WC, Adamus C (1997). Multicentric schwannomas causing chronic ruminal tympany and forelimb paresis in a Holstein cow. Vet Rec.

[18] Nielsen AB, Jensen HE, Leifsson PS (2011). Immunohistochemistry for 2′,3′-cyclic nucleotide-3′-phosphohydrolase in 63 bovine peripheral nerve sheath tumors. Vet Pathol.

[19] Quinn GC, Fews D, Scase TJ, Pearson GR (2005). Malignant peripheral nerve sheath tumour of the heart in a horse. Vet Rec.

[20] Kirchhof N, Scheidemann W, Baumgärtner W (1996). Multiple peripheral nerve sheath tumors in the small intestine of a horse. Vet Pathol.

[21] Rothwell TLW, Papadimitriou JM, Xu F-N, Middleto DJ (1986). Schwannoma in the testis of a dog. Vet Pathol.

[22] Pumarola M, Añor S, Borràs D, Ferrer I (1996). Malignant epithelioid schwannoma affecting the trigeminal nerve of a dog. Vet Pathol.

